# BARCOSEL: a tool for selecting an optimal barcode set for high-throughput sequencing

**DOI:** 10.1186/s12859-018-2262-7

**Published:** 2018-07-05

**Authors:** Panu Somervuo, Patrik Koskinen, Peng Mei, Liisa Holm, Petri Auvinen, Lars Paulin

**Affiliations:** 1 Mathematical Biology Group, Department of Biosciences, FIN-00014 University of Helsinki, P.O.Box 65 Finland; 2 Holm Group, Institute of Biotechnology, FIN-00014 University of Helsinki, P.O.Box 56 Finland; 3 DNA Sequencing and Genomics Laboratory, Institute of Biotechnology, FIN-00014 University of Helsinki, P.O.Box 56 Finland

**Keywords:** Barcode, DNA, Integer programming, Multiplexing, Optimization, Sequencing

## Abstract

**Background:**

Current high-throughput sequencing platforms provide capacity to sequence multiple samples in parallel. Different samples are labeled by attaching a short sample specific nucleotide sequence, barcode, to each DNA molecule prior pooling them into a mix containing a number of libraries to be sequenced simultaneously. After sequencing, the samples are binned by identifying the barcode sequence within each sequence read.

In order to tolerate sequencing errors, barcodes should be sufficiently apart from each other in sequence space. An additional constraint due to both nucleotide usage and basecalling accuracy is that the proportion of different nucleotides should be in balance in each barcode position. The number of samples to be mixed in each sequencing run may vary and this introduces a problem how to select the best subset of available barcodes at sequencing core facility for each sequencing run. There are plenty of tools available for de novo barcode design, but they are not suitable for subset selection.

**Results:**

We have developed a tool which can be used for three different tasks: 1) selecting an optimal barcode set from a larger set of candidates, 2) checking the compatibility of user-defined set of barcodes, e.g. whether two or more libraries with existing barcodes can be combined in a single sequencing pool, and 3) augmenting an existing set of barcodes.

In our approach the selection process is formulated as a minimization problem. We define the cost function and a set of constraints and use integer programming to solve the resulting combinatorial problem. Based on the desired number of barcodes to be selected and the set of candidate sequences given by user, the necessary constraints are automatically generated and the optimal solution can be found. The method is implemented in C programming language and web interface is available at http://ekhidna2.biocenter.helsinki.fi/barcosel.

**Conclusions:**

Increasing capacity of sequencing platforms raises the challenge of mixing barcodes. Our method allows the user to select a given number of barcodes among the larger existing barcode set so that both sequencing errors are tolerated and the nucleotide balance is optimized. The tool is easy to access via web browser.

**Electronic supplementary material:**

The online version of this article (10.1186/s12859-018-2262-7) contains supplementary material, which is available to authorized users.

## Background

It is a common practice to pool several samples together in order to maximize the usage of the capacity of high-throughput sequencing platforms. For example, at the moment, a single lane of Illumina HiSeqX produces hundreds of millions reads per run and the new NovaSeq can produce billions of sequences per run. If application requires only few tens of millions of reads per sample, it would be waste of resources to allocate an entire lane for a single sample. Therefore, several sequencing libraries are pooled together and sequenced in parallel using the same lane in the sequencing apparatus. This introduces the problem how to separate different samples after sequencing. A standard solution is to use a short barcode sequence for labeling different samples. These barcode sequences are attached to the fragments during the library preparation. The two processes, mixing the samples and then separating them after sequencing are also called multiplexing and demultiplexing, respectively.

In order to work properly, barcode sequences should be sufficiently different from each other. Redundancy in the barcode sequence provides the possibility for error correction. For example, in order to tolerate a single nucleotide mismatch in barcode detection, different barcode sequences should be at least three nucleotide mismatches apart from each other. More generally, in order to tolerate *m* mismatches, the distance between all barcode pairs should be at least 2*m*+1. Sequencing technology may give further restrictions for barcodes being optimal. For example, in Illumina sequencers, the nucleotides are detected using two lasers, red laser for A/C and green laser for G/T. For optimal detection, these two nucleotide groups should be in balance between all barcodes in each barcode position. Experiments show that reduced diversity in nucleotide composition results in data loss [1]. Besides nucleotide diversity being important in cluster identification, obtaining a good nucleotide balance is important for successful basecalling to be performed.

De novo barcode design, i.e. the process where the set of barcodes is constructed from scratch, is a solved problem and several tools are available for it, e.g. [2–4]. One of the first barcode designs was [5], where Hamming distance was used to measure the dissimilarity between the barcodes. Hamming distance has also been used in [6]. Taking into account insertions and deletions results in Levenshtein distance (also called edit distance), see e.g. [7]. Sequence similarity, complexity, GC content, and self-hybridization are taken into account in [8] and [9] includes nucleotide balance between barcodes which is important especially when multiplexing small number of samples using Illumina platform.

However, none of the aforementioned tools are applicable in the situation where the user wants to select an optimal set of barcodes among the existing barcodes. The only tool [4] which reports to do subset selection does it so that the user cannot even define the number of resulting barcodes and furthermore, the nucleotide balance is not taken into account, see Additional file 1. Selecting the subset randomly among the larger set of candidate barcodes has the assumption that all barcode subsets are equal. This is not the case since although the criterion for the minimum pairwise distance would be satisfied, different subsets have different nucleotide balances. In a sequencing center, the barcode selection is a practical daily problem. It would be waste of resources to order a unique set for each individual experiment. In the other extreme, if the same set of barcodes should be re-used in all future sequencing runs, in order to retain the nucleotide balance, the number of samples to be multiplexed should remain the same in all sequencing runs which would be highly restrictive.

At the moment, Illumina provides tables giving instructions how to select its own barcodes for multiplexing with various number of samples [10]. In these recommendations, nucleotide balance is taken into account. However, the tables are for Illumina’s fixed set of barcodes. Our tool lets the user provide her own set of candidate barcodes to be selected from. After the user has defined how many barcodes are needed, the tool finds an optimal set which satisfies the threshold for minimum pairwise sequence distances and importantly, the nucleotide balance has been optimized.

**Fig. 1:**
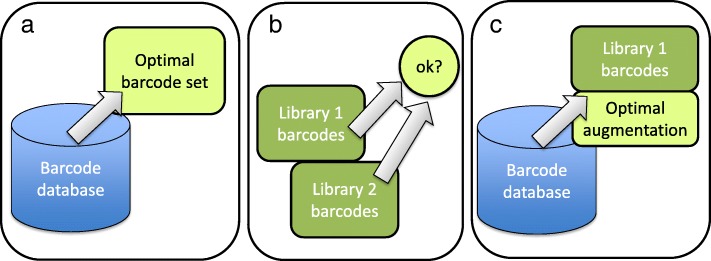
Applications of BARCOSEL. BARCOSEL can be used in three different modes depicted in panels **a**) selecting an optimal barcode set from candidates, **b**) checking user-defined set of barcodes, and **c**) augmenting an existing set of barcodes

Three tasks for which our tool can be applied are shown in Fig. 1. The main application is to select an optimal barcode set from given candidates (Fig. 1a). Another application is to check the barcode distances and nucleotide balance of user selected barcodes (Fig. 1b). For example, if there are two libraries, it can be used to check whether the barcodes in them are compatible to be sequenced together. Third application is to augment a set of barcodes (Fig. 1c). This is the case where the user wants to add new samples to an existing sequencing library. Optimal barcodes for new samples are found taking into account the already existing ones.

## Methods

Selecting a subset of barcodes among a set of candidate barcodes is a combinatorial problem. Let *x*_*i*_ denote the indicator for presence or absence of barcode *i*. The number of variables *x*_*i*_ is the number of all candidate barcode sequences given by user and they can get only binary values. The number of all possible subsets is restricted by the requirement of minimum distance between the barcodes. Further restrictions are introduced due to required nucleotide balance in the optimal set. The task is to select *n* barcodes among the set of user-defined candidate barcodes. After we have defined a cost function, we can use linear integer programming [11] for minimizing it. For optimal barcode selection, we define the following cost function to be minimized:

1 $$ \begin{aligned} {} \sum\limits_{l=1}^{L} \lvert n_{l}^{A} + n_{l}^{C} &- n/2 \rvert + \lvert n_{l}^{G} + n_{l}^{T} - n/2 \rvert \\[-4pt] &\quad+ \lvert n_{l}^{A} - n/4 \rvert + \lvert n_{l}^{C} - n/4 \rvert + \lvert n_{l}^{G} - n/4 \rvert \\&\quad\quad\quad\quad\quad\quad\quad\quad\quad\quad\quad\quad+ \lvert n_{l}^{T} - n/4, \rvert \end{aligned}  $$

where *L* is the barcode length and $n_{l}^{A}$, $n_{l}^{C}$, $n_{l}^{G}$, $n_{l}^{T}$ are the number of nucleotides A,C,G,T, respectively, in barcode position *l* in a selected set of *n* barcodes. This measures the nucleotide balance between the barcodes. The first two terms in (1) are for the balance of the two nucleotide groups for two Illumina lasers. The following four terms are for measuring the balance between single nucleotides. If all four nucleotides are in balance, also the nucleotide-pair groups are in balance and the first two terms are not needed in the cost function. However, if no perfect nucleotide balance can be found, the two first terms are important since they guide the solution towards the balance between the A/C and G/T groups. In addition to terms in (1), the final cost function includes also four terms for global nucleotide balance between A,C,G, and T irrespective of their positions. The number of different nucleotides $n_{l}^{\{A,C,G,T\}}$ in a selected barcode set is calculated using the barcode sequences with the help of indicator variables *x*_*i*_.

We have three types of constraints when minimizing the cost function. Since there are absolute differences in the equation, they must be formulated suitably for linear programming. Here we utilize the fact that min|*x*| corresponds to min*t* so that *t*≥*x* and *t*≥−*x*, where *t* is an auxiliary variable. For each nucleotide position in a barcode, there are six terms with absolute differences. We apply the method above to each of them and introduce six auxiliary variables for each barcode position. The values of the auxiliary variables are continuous, i.e., they are not restricted to be integers. For each auxiliary variable, there are two constraints. This way, if barcode length is eight nucleotides, there will be 48 auxiliary variables and 96 constraints. In addition, there are four auxiliary variables and eight constraints for the global nucleotide balance. It is noteworthy that the number of nucleotide balance constraints does not depend on the number of barcode candidates, it only depends on the length of the barcodes. The second type of constraints are for preventing too similar barcodes to be present in the optimal set. The similarity is measured using user-defined distance (Hamming or Levenshtein). If the distance between two barcodes *j* and *k* is below the given threshold, they both should not be present at the same time in the optimal barcode set. This constraint is formulated as *x*_*j*_+*x*_*k*_≤1. Forbidden barcode pairs are detected by calculating the distances between all barcodes sequences. Note that although here we use Hamming or Levenshtein distance, it is straightforward to use any other sequence distance. How the constraint is formulated to deny illegal barcode pairs remains the same regardless of the sequence dissimilarity function. The third type of constraints is a single equation. It defines the number of selected barcodes. In case the number of all candidate barcodes is *M* and the number of barcodes to be selected is *n*, the last constraint is $\sum \limits _{i=1}^{M} x_{i} = n$.

After generating the constraints described above, any integer programming solver can be used. We have integrated C-library lpsolve version 5.5 [12] in our software. User provides a set of candidate barcodes in a FASTA file and the desired number of barcodes to be selected. Output is a FASTA file which contains the selected barcodes and a graphical diagnostic plot which shows the position-wise and global nucleotide balance. Minimum barcode distance is a strict criterion, so if a solution is found, it is guaranteed that no barcode pair in a selected set is below the chosen threshold.

## Results

**Fig. 2:**
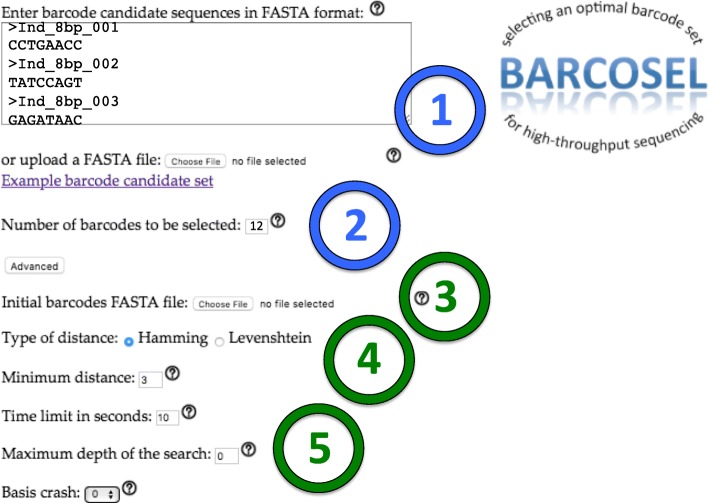
Web interface to BARCOSEL. User needs to give only two inputs: (1) Candidate barcodes in FASTA format and (2) the number of barcodes to be selected. Sequences can imported either by copy-pasting them in a text box or uploading a FASTA file. After user has pressed submit button, the web page is returned containing an optimal set of barcodes and an image showing nucleotide balance in each barcode position. In case no solution can be found, user gets a report. Optional input parameters become available when pressing Advanced button. These include (3) an initial set of barcodes if BARCOSEL is used to augment an existing set, (4) distance type (Hamming or Levenshtein) and minimum barcode distance required (default is 3 tolerating one sequencing error), and (5) parameters related to lpsolve: maximum computation time (default is 10 seconds), branch-and-bound search depth (0 means no restrictions), and basis crash parameter related to initialization. There is no need to change lpsolve related parameters unless no acceptable solution is found with default values

Our tool is available through web interface, see Fig. 2. User can give a set of candidate barcodes in FASTA format and the number of barcodes to be selected. In order to help getting started, our web page contains an example set of 288 barcodes which can be used to select barcodes from. The user can define a minimum distance between barcodes using either Hamming distance (number of nucleotide differences between two barcodes in the gapless alignment) or Levenshtein distance (number of substitutions, insertions, and deletions to convert one barcode sequence into another). The default minimum barcode distance is 3 using Hamming distance. When user wants to only check the nucleotide balance in an existing barcode set and whether the barcode distances exceed the given threshold value, the number of barcodes to be selected can be defined to be equal to the total number of barcodes in the input set. When user wants to expand an existing subset of barcodes, the initial subset is provided using Advanced menu. The desired number of new barcodes which optimally expand the existing subset is selected from candidate barcodes.

We have applied our method to a barcode set consisting of 288 candidate sequences having a length of 8 nucleotides. These barcodes were initially designed using TagGD software [8] and further screened to be suitable for PCR [13]. We have used this set for selecting various number of barcode sets for different purposes. Some examples are shown in Table 1. The best possible position-wise nucleotide balance depends on the number of barcodes to be selected. Only when it is a multiple of four, there can be an equal number of A, C, G, and T in every barcode position resulting in perfect balance and cost function equal to zero. In this case also the solution is found fast, usually within only few seconds. In other cases, when the best solution differs from zero, even when the global optimum has been found, the search process will continue. For this reason, there is a user-defined time limit to stop the search. When it exceeds, the best solution found so far is returned. With our data, less than 20 s was always enough to get a satisfactory solution. Additional user-defined parameters include the maximum depth of branch and bound search. If acceptable solution is not found, it might be beneficial to widen the search at the cost of its depth. Another parameter is the initialization method of the search which can be changed.

**Table 1:** Examples of optimal 8bp barcode sets with 8,12,16, and 24 barcodes

Set A (8)	Set B (12)	Set C (16)	Set D (24)
AACACATC	AGTTGCTG	ACACAGGC	ACACAGGC
AGAGTGCG	ATAGAGTC	ACTGTTAG	AGCCTACT
CCGTATAT	ATCATTGC	ATAGAGTC	AGTTCCGC
CTTGGTTG	CAGTTCCA	ATTAGCTG	ATACGGAT
GAGATAAC	CATGGAAT	CAGTTCCA	ATGACGAA
GTACAGGA	CGCAAGCT	CCGTATAT	ATGGTCTC
TCTCGCCT	GAGATAAC	CGCAAGCT	CAGTTCCA
TGCTCCGA	GCAGATAA	CTGGCACA	CATGTTGA
	GGCTCTTG	GACACTAA	CCTGAACC
	TCGCCAGA	GGAGTAGA	CGAACTTC
	TCTCGCCT	GGCTCTTG	CTCCGGTT
	TTACCGGG	GGGAGATC	CTGAATCA
		TACTGCAG	GAAAGAAG
		TATCCAGT	GAGATTGT
		TCTCGCCT	GCATCACG
		TTACTGGC	GCCGAATG
			GCTGAAGA
			GGCTCTTG
			TACTGCAG
			TATCTGTG
			TCTCGCCT
			TGAGAGAT
			TGCTCCGA
			TTGAGTAC

Each barcode is at least three mismatches apart from each other (using Hamming distance) within the set allowing one nucleotide error in sequencing to be corrected. Proportions of all four nucleotides A,C,G,T are in balance in each barcode position

**Fig. 3:**
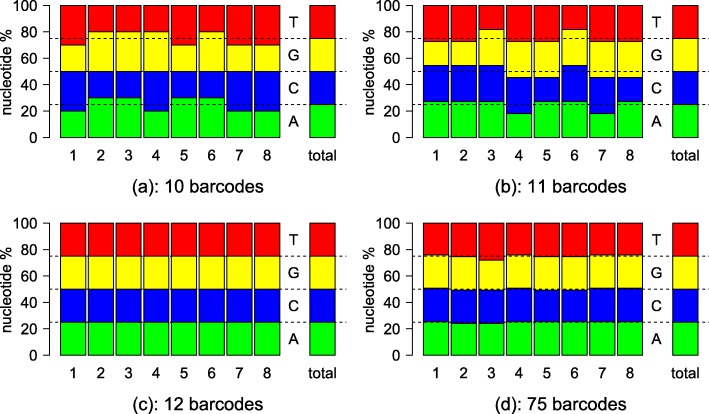
Nucleotide balances of optimal sets with varying number of barcodes. Horizontal axis is the barcode position, total indicates the nucleotide balance over the entire barcode length. In Illumina sequencing, nucleotide groups A/C and G/T should be in balance for optimal detection in each position. Total balance is important for equal consumption of nucleotides during sequencing. **a** 10 barcodes. **b** 11 barcodes. **c** 12 barcodes. **d** 75 barcodes

Figure 3 shows examples of four optimal barcode sets. When the number of barcodes in the set is even, it is possible to get perfect balance between A/C and G/T groups in every barcode position (Fig. 3a). When the number is odd, this is not possible, however, it is still possible to get perfect nucleotide balance over the barcode length, see the rightmost column in Fig. 3b. This means that the nucleotide usage between A,C,G, and T will be the same in sequencing, at least for the barcode. When the number of barcodes is a multiple of four, it is possible to get perfect nucleotide balance in every barcode position (Fig. 3c). If the number of barcodes is large, it is possible to get near perfect balance even when the number of barcodes is odd (Fig. 3d).

## Discussion

Although in principle there are no restrictions for the size of the data the method can handle, in practice the computation time grows when the size of the input barcode set increases. For a practical advice, our tool is mostly applicable for selecting a subset of barcodes from an existing larger set which does not exceed several thousands of candidates. In particular, our tool is not meant to be used in the situation where the user first enumerates all possible 8-mers (65,536 barcodes) and then starts selecting a subset of it. For this kind of application it is better (faster) to use de novo barcode design tools. We have mainly used our tool for RNA-seq and genome re-sequencing, where the number of libraries to be multiplexed varies between 10 and 20, and the size of the input data consists of a few hundreds of barcode candidates, see e.g. [14]. The optimization of nucleotide balance is most important with small number of samples. When the number of libraries to be multiplexed becomes larger and therefore larger number of barcodes are used together, the nucleotide balance will become eventually evenly distributed even by chance. In such cases it suffices to check that the sequence distances (measured by Hamming or Levenshtein distance) between all barcode pairs are adequate.

Finally, for large number of multiplexed samples, the method of dual indexing can be used. In this method two different barcodes are attached to each sample. For example, if there is a need to multiplex 384 samples, it can be done as a combination of 24-barcode P7 index set and 16-barcode P5 index set (24∗16=384).

## Conclusions

Increasing capacity of sequencing platforms raises the challenge of mixing barcodes during the protocols in RNA-seq, whole genome sequencing, and amplicon sequencing approaches. The number of samples to be mixed may vary which introduces a problem how to select the best barcode combination for each sequencing run. Instead of designing a new barcode set from scratch for each sequencing run, the practical problem is how to select the best combination of barcodes from available existing set of barcodes. This is the task we have developed our method for. Our tool selects the desired number of barcodes in such a way that the nucleotide balance of barcodes is optimized. In addition, user can set a minimum distance between barcodes to tolerate sequencing errors. We have successfully used our method in several sequencing projects of various kinds of assays. Web interface to our tool is available at http://ekhidna2.biocenter.helsinki.fi/barcosel. It contains instructions and an example candidate barcode set to be used for subset selection.

## Additional file

Additional file 1 Barcode selection using BARCOSEL and R-package DNABarcodes. (PDF 78 kb)
