# Supplementary material for: BARCOSEL: a tool for selecting an optimal barcode set for high-throughput sequencing
Source: BMC Bioinformatics. 2018 Jul 5;19:257. doi: 10.1186/s12859-018-2262-7 (PMC6034344; doi:10.1186/s12859-018-2262-7)
Supplement: Supplementary file 1 — Barcode selection using BARCOSEL and R-package DNABarcodes. (PDF 78 kb) [file 12859_2018_2262_MOESM1_ESM.pdf]

Supplement

The following demonstrates the barcode subset selection using BARCOSEL and R-package DNABarcodes. At the time of writing the manuscript, these were the only tools available to allow user to do subset selection from the pool of user-defined candidate barcodes. In order to favor DNABarcodes, the initial barcode set was created using its de novo design process. It is worth noting that with DNABarcodes user cannot define the number of barcodes to be returned. This applies to both its de novo barcode design and subset selection (situation June 2018). We started by creating barcodes with the length of 4bp using the following R commands:

```
> library(DNABarcodes)
> pool1=create.dnabarcodes(4)
```

This resulted in 12 barcodes. A prefix "CA" was added to each of them and in addition, four barcodes were copied and their prefix was set to be either "GC", "AG", "CT", or "TA". The final candidate barcode set consisted of 16 barcodes with the length of 6bp stored in file "pool2.txt" (each barcode in a separate line):

```
CAGGAA, CACAGA, CAACCA, CATCAG, CAATGG, CAAATG, CACTAC, CATACC, CAAGTC, CATGGT,
CAGTCT, CACCTT, GCATGG, AGTACC, CTGGAA, TACCTT
```

In this example the goal was to select 4 barcodes from the set of 16 candidates, but there is no input parameter in DNABarcodes to set the number of barcodes to be returned.

```
> pool2=as.character(read.table("pool2.txt")[,1])
> subset=create.dnabarcodes(6,pool=pool2)
```

DNABarcodes selected the following 11 barcodes as a subset:

```
CAGGAA, CACAGA, CAACCA, CATCAG, CAATGG, CACTAC, CATACC, CAAGTC, CATGGT, CAGTCT,
CACCTT
```

There is no variation in the first two nucleotide positions. The result was the same when using any of the four heuristics: “conway”, “clique”, “sampling”, and “ashlock”. When BARCOSEL was used to select four barcodes from the same 16-barcode candidate set, it gave the following four barcodes:

```
GCATGG, AGTACC, CTGGAA, TACCTT
```

Figure below shows the nucleotide balance in the two subsets. Even when using any selection of four barcodes from the subset of 11 barcodes which DNABarcodes returned, there could be no variation in the two first nucleotide positions. This demonstrates that DNABarcodes does not take nucleotide diversity nor balance into account. The subset selected by DNABarcodes in this example could not be used for multiplexing samples if they are going to be sequenced by Illumina platform.

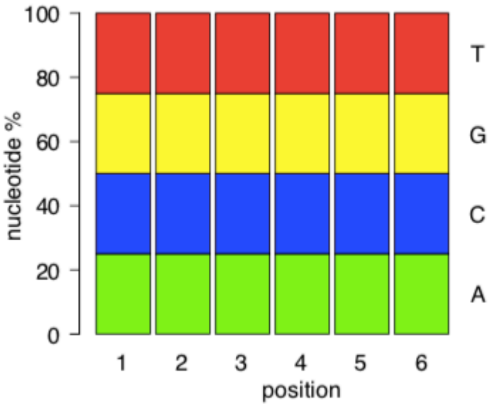

(a): BARCOSEL

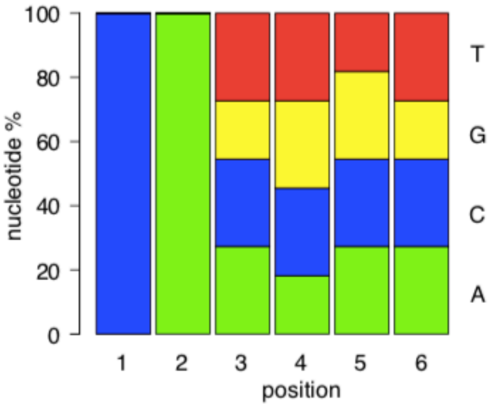

(b): DNABarcodes
